# Case report: Spinal cord stimulation for pain relief in two patients with locally recurrent pelvic malignancy

**DOI:** 10.3389/fonc.2024.1403703

**Published:** 2024-06-24

**Authors:** Yousif Salem, Charles T. West, Malcolm West, Hideaki Yano, Paul Fernandes, Girish Vajramani, Alexander Mirnezami

**Affiliations:** ^1^ Southampton Complex Cancer and Exenteration Team, University Hospitals Southampton NHS Foundation Trust, Southampton, United Kingdom; ^2^ Cancer Sciences, Academic Surgery, Faculty of Medicine, University of Southampton, Southampton, United Kingdom; ^3^ Department of Anaesthesia, University Hospitals Southampton NHS Foundation Trust, Southampton, United Kingdom; ^4^ Centre for Functional Neurosurgery, Department of Neurosurgery, University Hospitals Southampton NHS Foundation Trust, Southampton, United Kingdom

**Keywords:** locally recurrent anal cancer, locally recurrent rectal cancer, pain relief, palliative therapy, spinal cord stimulation

## Abstract

**Introduction:**

Chronic cancer-related pain from locally recurrent infiltrative cancers within the bony confines of the pelvis is a devastating and hard to manage condition that can be refractory to many conventional pain management methods. Spinal cord stimulation (SCS) is an evolving and safe method of pain management and can be trialled in a quick and well-tolerated operation under local anaesthesia. To date, this has not been reported in the setting of locally recurrent inoperable pelvic cancers.

**Case description:**

In the present study, we report two cases of patients with severe back and lower limb pain resulting from recurrent anal and rectal cancers involving the right lumbar and sacral nerve roots as well as the bony sacrum, which severely affected quality of life and daily functioning.

**Discussion:**

Following successful SCS, effective pain relief was observed.

**Conclusion:**

SCS could represent an effective supplementary or alternative technique to conventional pain management in this challenging group of patients, especially if other available methods have been exhausted.

## 1 Introduction

Cancer-related pain is a significant public health issue facing patients with cancer worldwide; it affects over one-third who are curatively treated, half who are still under treatment and up to two-thirds with terminal, advanced, or metastatic disease. Moderate to severe pain is self-reported by more than one-third of patients with cancer, and evidence suggests that undertreatment remains a significant issue for many, leading to emotional distress, impaired quality of life, and disability (1–3).

Locally recurrent and advanced pelvic cancers represent a heterogenous group of diseases that share a significant burden of pain. While multimodality treatment incorporating radical surgical resection remains the cornerstone of patient management, symptom control can be exceptionally challenging for inoperable patients, with intractable pelvic pain often refractory to opiates being one of the key symptoms (4–6).

Pelvic pain in such tumours is typically a consequence of a combination of a growing pelvic mass within the confines of the bony pelvis, and direct cancer invasion of pelvic nerves, bones, and muscles.

Spinal cord stimulation (SCS) is a form of neuromodulation increasingly applied for the treatment and control of chronic neuropathic pain from a variety of aetiologies (7). The technique involves the initial radiologically guided insertion of a temporary electrode into the epidural space which is then steered to the desired anatomical location. If stimulation of this trial electrode achieves successful pain relief, then a permanent implantation is conducted. The applications of SCS are increasingly evolving and common examples where it has gained acceptance include the management of chronic back pain, lower limb mononeuritis, spinal stenosis, critical limb ischaemia, and complex regional pain syndromes (8). To date, however, SCS has not been applied for the palliative management of cancer-related pain. In a national review of over 12000 cases of SCS, management of pelvic cancer pain was not one of the indications identified (8). A systematic literature review identified the use of SCS in 56 patients with cancer, with only 3 found to have cancer involving the pelvis, and this as a consequence of metastasis to the spine from renal, prostate, and thyroid cancers (9, 10). To date, the application of this modality to locally recurrent pelvic cancers has not been described. Herein, we present two cases of successful management of chronic pain secondary to local recurrence of pelvic cancers using SCS as a palliative strategy.

## 2 Case description

### 2.1 Ethical considerations

Written informed consent was obtained from the patients described in this study.

### 2.2 Case 1

**Figure 1:**
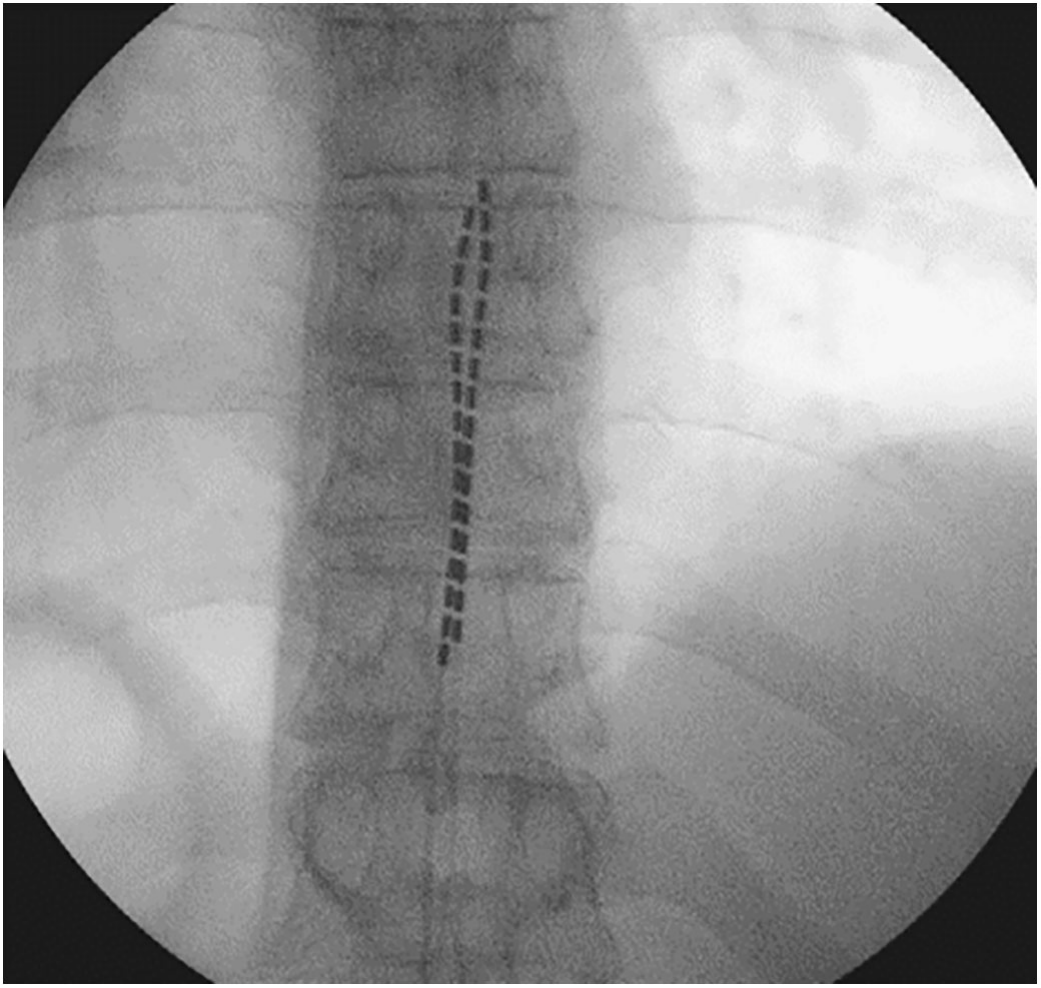
Location of permanent spinal cord implantation at the T9 – T11 level.

The patient was a 54-year-old man with a history of anal squamous cell carcinoma diagnosed in 2017 and treated with radical chemo-radiotherapy (50.4 Gy). Following a local recurrence in 2019, he underwent an extra-levator abdominoperineal excision and vertical rectus abdominis muscle (RAM) flap reconstruction. Subsequently, he developed a further recurrence in 2021, with an extensive pelvic tumour involving the base of the penis, both inferior pubic rami, the lower edge of the symphysis pubis, the rectus abdominis muscle (RAM) flap, and the right sacral ligaments. Following a course of neoadjuvant/palliative intent chemotherapy, he underwent a total infralevator pelvic exenteration with en bloc distal sacrectomy, resection of the right ischial spine, right ischial tuberosity, and bilateral inferior ischiopubic rami, and partial resection of the pubic symphysis and perineal reconstruction with a combination of flaps and bovine acellular dermis. An R0 resection was achieved, and the patient achieved a good recovery with postoperative scans showing no signs of any residual disease. Subsequently, in January 2023, further re-recurrence was suspected radiologically involving the right sciatic nerve and a groin lymph node and confirmed histologically by percutaneous biopsy. The patient initially underwent a course of External Beam Re-irradiation (30.6 Gy). Upon completion of re-irradiation, the pain intensity in the patient’s right lower limb and perineum increased progressively and was found to be refractory to combinations of high dose opiates, non-steroidal drugs, and gabapentinoids. The pain intensity was rated at 8–9 (out of 10) and described as being sharp and continuous, although partially relieved by analgesia, resulting in impaired sleep quality and an inability to walk and drive. Various alternative options were attempted, including palliative chemotherapy with Carboplatin and Paclitaxol, extensive physiotherapy, acupuncture, and transcutaneous electrical nerve stimulation, but to no avail. After a multidisciplinary team discussion with palliative care and neurosurgical teams regarding pain control, a decision to trial SCS was offered and accepted by the patient (**Figure 1**). He underwent implantation of a thoracic percutaneous permanent SCS with on-table testing and an implantable pulse generator (x2 Infinion CX leads, WaveWriter Alpha, Boston Scientific). Substantial improvement was observed on table and in the post-operative period in terms of pain control and quality of life utilising the novel fast-acting sub-perception therapy (FAST) (Boston Scientific), with the patient describing the change as ‘dramatic’ and ‘like flicking a switch.’ The base FAST programme was set at 35% with a regular bolus every 4 hours at 70% for 30 minutes. The patient stated that he could sleep continuously at night for at least 4 h without experiencing pain, walk using a stick, and drive short distances. Additionally, the doses and frequencies of his analgesics were significantly reduced. No complications of the SCS were noted 6 months post-implantation.

### 2.3 Case 2

**Figure 2:**
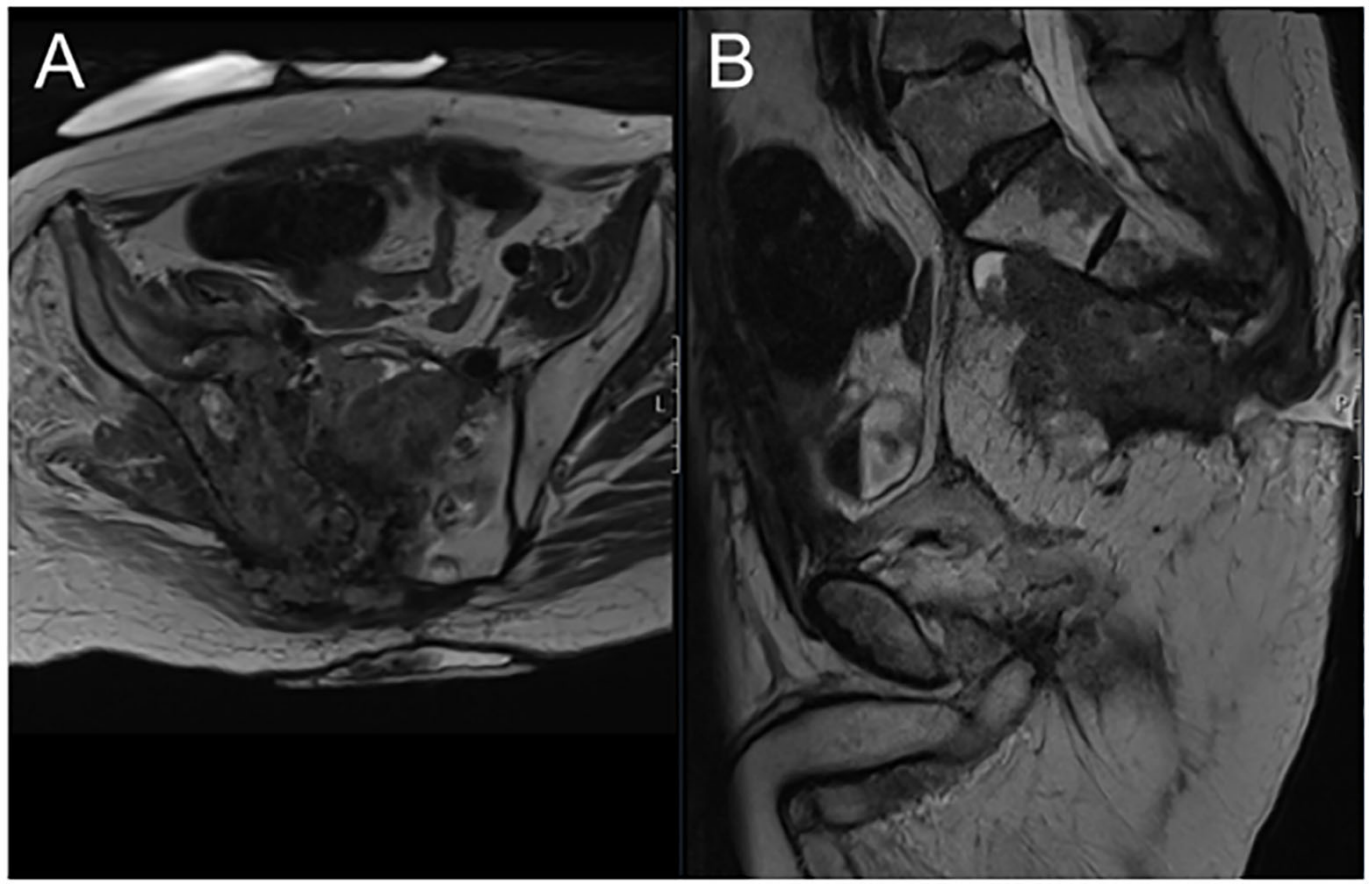
Axial MRI view showing locally recurrent tumour mass invading the right sacro-iliac joint and replacing the sacral body **(A)** sagittal MRI view of the same patient **(B)**.

The patient was a 71-year-old man, who was treated for rectal cancer initially with a laparoscopic anterior resection in 2014. While on a postoperative surveillance programme, he underwent CT and magnetic resonance imaging of the abdomen and pelvis in 2016, which showed a growing soft tissue mass on the right side of the pre-sacral space. The mass was biopsied and confirmed as a local recurrence. Subsequently, he underwent neoadjuvant chemotherapy with FOLFOX followed by neoadjuvant chemoradiotherapy (50.4 Gy in 25 fractions) followed by total pelvic exenteration with high sacrectomy, left pelvic sidewall resection and Intraoperative electron beam radiotherapy (12 Gy boost) to the left pelvic sidewall. The final histopathological results showed R0 resection of a moderately well differentiated adenocarcinoma and two separate foci of adenocarcinoma at the previous peri-anastomotic level. The patient was enrolled in a surveillance programme until April 2018, when follow-up imaging revealed local recurrence along the contralateral right sacro-iliac joint. Initial palliative chemotherapy and IMRT re-irradiation (30.6 Gy in 17 fractions) did not improve the situation and subsequent cryo-ablation of the pre-sacral mass was performed in December 2018 for symptom and pain control. However, the patient developed severe lower limb pain fluctuating between 6 and -10/10 on the numerical rating scale, starting from the right buttock and radiating to his toes, and significantly affecting his quality of life. Right leg weakness and foot drop from the involvement of the sciatic nerve were noted (**Figure 2**). Despite high doses of opiates and gabapentinoids, pain was poorly controlled. Thus, after a multidisciplinary team discussion with oncology and pain management and neurosurgical teams, SCS was considered and offered. The SCS was implanted in January 2021. Thoracic percutaneous leads and a non-rechargeable implantable pulse generator (x2 Octrode, Proclaim 5 Elite IPG, Abbott Medical) were implanted. On-table testing revealed excellent paraesthesia coverage of the pain areas. Programming was performed with burstDR as well as a paraesthesia-based programme. At follow-up, the patient reported that the right lower limb pain had completely disappeared. He was followed up for 8 months during which time his self-reported pain was significantly improved (opioid analgesic requirements halved). However, he finally succumbed to progressive cancer. No complications from the SCS were noted during this time.

## 3 Discussion

Although opioids are the mainstay of treatment in many patients with cancer-related pain, it is a well described observation that patients with locally recurrent pelvic cancers often have severe pain refractory to opioids (5, 6). Moreover, patients often describe significant opioid-related side effects. Although the addition of gabapentinoids to the management of such patients has provided some benefit, there is a not insignificant group of patients who fail to obtain symptomatic relief from pain with such combination therapies (5, 6).

As minimally invasive neuromodulator devices and techniques that effectively manage and treat chronic cancer pain continue to emerge, the popularity of SCS as a bespoke option in pain management is increasing, especially among patients who have exhausted all available methods (10). Although the mechanism of action of SCS in controlling pain remains elusive, it is known to interfere with the pathway of pain neuro-transmitters in the central and supraspinal centres and reduces the excitability of nociceptive fibres in the dorsal column of the spinal cord and spinothalamic pathways (11, 12). Previously, the most frequent SCS modalities used were low frequency paresthesia (case 2). In more recent times however, a FAST modality is increasingly applied which can produce a more rapid onset of pain relief and which does not elicit paresthesia (case 1).

A clear limitation of this report is the few patients described. In addition, it should be noted that high quality studies in this field are currently lacking, with no randomised studies on the use of SCS in chronic cancer related pain. Furthermore, there are drawbacks to the use of SCS which need to be considered. The overall complication rate from SCS placement in the non-cancer setting has been reported to be 31.9 - 43% with the most frequent complication being electrode migration. More serious complications such as spinal cord injury have also been described although with a low incidence rate of below 0.5%. Other complications include cerebrospinal fluid leaks, infections, haematoma formation, and more rarely, epidural fibrosis and syrinx formation (13). Nevertheless, despite such reported complications, in the setting of inoperable pelvic cancers with a mortality measured in months, carefully selected and counselled patients may benefit from this intervention with an improvement in their quality of life.

## 4 Conclusion

We describe here the first report of SCS use in patients with inoperable locally recurrent pelvic cancers. SCS may represent an effective addition or alternative for the treatment and control of refractory cancer-related pain from locally recurrent pelvic tumours.

## Data availability statement

The original contributions presented in the study are included in the article/supplementary material. Further inquiries can be directed to the corresponding author.

## Ethics statement

Written informed consent was obtained from the individual(s) for the publication of any potentially identifiable images or data included in this article.

## Author contributions

YS: Conceptualization, Writing – original draft, Writing – review & editing, Data curation, Investigation, Project administration. CW: Data curation, Investigation, Writing – review & editing. MW: Writing – review & editing, Supervision. HY: Writing – review & editing. PF: Writing – review & editing, Conceptualization. GV: Writing – review & editing, Conceptualization. AM: Conceptualization, Supervision, Validation, Writing – review & editing.
